# Encorafenib plus binimetinib in patients with BRAF-mutated melanoma brain metastases: Results from the BECARE (GEM-2002) trial

**DOI:** 10.1093/noajnl/vdag252

**Published:** 2026-09-21

**Authors:** Pablo Cerezuela-Fuentes, Miguel-Angel Berciano-Guerrero, Eva Muñoz-Couselo, Pedro Sanchez Mauriño, Regina García Galindo, Lourdes Gutiérrez Sanz, José Balsalobre Yago, Teresa Puértolas, Carlos Aguado, Silvia Sequero, Guillermo Crespo, Cristina Aguayo, Enrique Espinosa, Pedro López, Rafael López Castro, Joaquín Fra Rodríguez, Gretel Benítez, Alberto Jacobo Cunquero-Tomás, Alfonso Berrocal, Ainara Soria

**Affiliations:** Medical Oncology, Hospital Clínico Universitario Virgen de la Arrixaca, Instituto Murciano de Investigación Biosanitaria (IMIB)-Arrixaca, Ciudad de Murcia, Spain; Medical Oncology, Hospital Regional Universitario (HRU), Instituto de Investigaciones Biomédicas de Málaga-Plataforma BIONAND (IBIMA-BioNAND), Málaga, Spain; Medical Oncology, Hospital Universitario Vall d‘Hebron & Vall d‘Hebron Institute of Oncology (VHIO), Barcelona, Spain; Medical Oncology, Hospital Universitario Reina Sofia, Córdoba, Spain; Medical Oncology, Hospital Universitario de Jerez de la Frontera, Jerez de la Frontera, Spain; Medical Oncology, Hospital Universitario Puerta de Hierro, Majadahonda, Spain; Medical Oncology, Hospital General Universitario Santa Lucía, Cartagena, Spain; Universidad Católica de Murcia (UCAM), Spain; Instituto Murciano de Investigación Biosanitaria (IMIB), Murcia, Spain; Medical Oncology, Hospital Universitario Miguel Servet, Zaragoza, Spain; Medical Oncology, Hospital Universitario Clínico San Carlos, Madrid, Spain; Medical Oncology, Hospital Universitario San Cecilio, Granada, Spain; Medical Oncology, Hospital Universitario de Burgos, Burgos, Spain; Medical Oncology, Hospital Universitario Infanta Sofía, San Sebastián de los Reyes, Spain; Universidad Autónoma de Madrid, School of Medicine—Hospital Universitario La Paz—CIBERONC—Madrid, Madrid, Spain; Medical Oncology, Hospital Universitario de Jaén, Jaén, Spain; Medical Oncology, Hospital Clínico Universitario de Valladolid, Valladolid, Spain; Medical Oncology, Hospital Universitario Río Hortega, Valladolid, Spain; Medical Oncology, Complejo Hospitalario Universitario Insular-Materno infantil de Gran Canaria, Las Palmas de Gran Canaria, Spain; Medical Oncology, Consorcio Hospital General Universitario de Valencia, Valencia, Spain; Medical Oncology, Consorcio Hospital General Universitario de Valencia, Valencia, Spain; Medical Oncology, Hospital Universitario Ramón y Cajal, Madrid, Spain

**Keywords:** binimetinib, encorafenib, melanoma, melanoma brain metastasis, targeted therapy

## Abstract

**Background:**

Encorafenib plus binimetinib (EB) is a standard treatment for BRAF^V600^-mutated melanoma brain metastasis (MBM). However, there is limited evidence of EB use in MBM from clinical trials, and real-world data are scarce. This study aimed to determine the effectiveness and safety of EB in BRAF^V600^-mutated MBM.

**Methods:**

BECARE-GEM-2002 was a retrospective study conducted in 24 hospitals in Spain, including patients treated according to standard clinical practice with EB as the 1st line or 2nd line of treatment after progression to immunotherapy, regardless of symptoms and corticosteroid use. Patients had measurable intracranial disease (≥5 mm on CT scan). Previous treatment with BRAF/MEK inhibitors was not allowed.

**Results:**

Between 2021 and 2025, 60 patients were included, 52 (86.7%) treated upfront and 8 (13.3%) after immune checkpoint inhibitors (ICIs) progression. The ORR was 58.3% (95% confidence interval [CI], 44.9-70.9), 65.4% (95% CI, 44.3-82.8) in asymptomatic, and 52.9% (95% CI, 35.1-70.2) in symptomatic MBM. Radiotherapy (RT) was administered prior to EB in 7 patients (11.7%) and concurrently in 17 (28.3%). Patients receiving RT showed an ORR of 76% (95% CI, 54.9-90.6). The median PFS was 7.4 months (95% CI, 6.2-9.7) and median OS was 10.6 months (95% CI, 9.2-15.0). Treatment-related adverse events of grade ≥3 were reported in 12 patients (20%), the most frequent being transaminitis (6.7%) and gastrointestinal hemorrhage (3.3%).

**Conclusions:**

EB was safely administered in the real world and showed clinically significant benefit in patients with BRAF^V600^-mutant MBM, regardless of brain symptoms. The potential role of RT should be explored in prospective trials.

Key Points

- EB showed real-world effectiveness in BRAF^V600^-mutated melanoma brain metastasis, particularly in patients with corticosteroid dependency.
- EB has a manageable safety profile.
- Brain radiotherapy before or during EB treatment showed improved antitumour activity.

Importance of the Study

This study provides further evidence for the use of encorafenib plus binimetinib in real-world patients with melanoma brain metastasis, which is an unmet clinical scenario. The results support the use of encorafenib plus binimetinib in patients with BRAF-^V600^-mutated melanoma brain metastasis, especially in patients with symptoms or corticosteroid dependency, and encourage prospective exploration of the potential role of adding radiotherapy (RT).

The incidence of melanoma has been rising in the last decades, accounting for 19/100.000 people in western Europe^1^ and over 40% of patients eventually develop melanoma brain metastases (MBMs) during the disease course.^2^ Despite the rise of new therapeutic strategies for advanced/metastatic disease, including targeted therapy and immune checkpoint inhibitors (ICI), the management of MBMs is particularly challenging, and the prognosis remains dismal.^3^^,^^4^

Dual ICI with ipilimumab/nivolumab has been positioned as the preferred first-line systemic therapy for patients with BRAF-native asymptomatic MBMs.^5-9^ For patients with BRAF^V600^-mutated MBM, treatment with BRAF/MEK inhibitors (i) is also available,^10-14^ although the best treatment sequence or personalized medicine strategies have not been clearly defined. Several studies have reported that a sequence of ICI followed by BRAF/MEKi or a sandwiched ICI between BRAF/MEKi may protect against the appearance of brain metastasis,^15^^,^^16^ however, no clear preference is established once brain metastases were present. Upfront BRAF/MEKi followed by ICI is a viable strategy for symptomatic MBMs, depending on the extension and symptoms of the disease.

Most phase III studies excluded patients with symptomatic brain metastases, complicating the translation of results from these trials to this particular population. Studies including patients with symptoms of brain metastasis or dependence on corticosteroids at the initiation of systemic therapy are challenging due to the limited incidence and their poor prognosis.

Encorafenib plus binimetinib showed favorable efficacy compared with vemurafenib in the phase 3 COLUMBUS trial.^17^ However, only nine patients included in the COLUMBUS trial had MBM. Some small studies proved encorafenib plus binimetinib has promising efficacy in MBM.^11^^,^^12^ The sequential combination of encorafenib plus binimetinib and intracranial radiotherapy for MBM yielded an intracranial response rate (icORR) of 70.8%, with 10.4% of patients showing a complete response (CR).^12^ Results from real-world data on encorafenib plus binimetinib have reported promising clinical benefits in MBM,^18^^,^^19^ but evidence is scarce, and no real-world data are available for the Spanish population.

Given the limited evidence of encorafenib plus binimetinib treatment in MBM, particularly in symptomatic brain metastases or after progression to ICI, real-world evidence is of utmost relevance. In this context, we leveraged data from the real-world BECARE study to determine the effectiveness and safety of encorafenib plus binimetinib in patients with BRAF^V600^-mutated MBM.

## Methods

### Study Design and Objectives

The BECARE study (GEM 2002)^20^ was a multicenter, retrospective, non-interventional, observational study aimed at exploring the efficacy and safety of encorafenib plus binimetinib in patients diagnosed with unresectable or metastatic (TNM Classification, 8th edition) BRAF^V600^-mutant melanoma. The secondary objectives were to describe the baseline demographic and pathological characteristics of patients with BRAF^V600^-mutated MBM treated with encorafenib plus binimetinib in Spain, their treatment compliance, survival outcomes, and the influence of prognostic factors and prior systemic treatments on effectiveness. This manuscript comprises a subset analysis on the cohort of patients with MBM, the BECARE-BRAIN substudy.

The study was conducted in 24 hospitals in Spain. All patients with melanoma identified between September 2021 and December 2025 at the participating hospitals who met the eligibility criteria were included to mitigate selection bias. The study retrieved data from the patients’ medical records retrospectively; therefore, therapeutic decisions were made before and independently from inclusion in the study, following standard clinical practice. Data from the patients’ clinical registries were collected by trained data managers and investigators for at least 12 months.

This study was registered with the Spanish Registry of Clinical Studies (Ref: 0004-2021-OBS) in May 2021. All patients alive were requested to provide informed consent prior to study entry. The ethics committee granted an exemption from informed consent for patients who died before the study was initiated.

### Patients

The study enrolled patients with advanced or metastatic BRAF^V600^-mutated melanoma treated with 450 mg of encorafenib once daily and 45 mg of binimetinib twice daily orally, according to the standard indications in the summary of product characteristics (SmPC). Encorafenib plus binimetinib must have been administered as first- or second-line treatment after progression to ICIs for advanced or metastatic disease. After the publication of the initial results,^20^ the study was expanded to include more patients with MBM who were treated with encorafenib plus binimetinib. Patients were required to have measurable intracranial disease (at least one lesion of 5 mm or one lesion smaller than 5 mm measurable detected by magnetic resonance imaging [MRI]). Both asymptomatic and symptomatic MBM cases were eligible, regardless of corticosteroid use. Symptom determination relied on real-world clinical documentation, based on the clinical judgment of the treating physicians that considered central nervous system (CNS)-relatedness, temporary association with the brain disease diagnosis/progression, its associated edema or requirement of clinical interventions such as corticosteroid initiation. Patients who had previously received treatment with another BRAF and/or MEK inhibitor were excluded, except for management of resected locoregional disease (ie adjuvant setting) ≥ 6 months before initiation of encorafenib plus binimetinib.

### Endpoints

Demographic and disease attributes were collected to characterize the target population receiving encorafenib plus binimetinib. Efficacy endpoints comprised objective response rate (ORR), defined as the percentage of patients that achieve partial response (PR) or CR as their best responses per Response Evaluation Criteria in Solid Tumors (RECIST) v1.1; duration of response (DoR), defined as the time elapsed from the first determination of response (PR or CR) to the progression of the disease or death, whichever occurs first; disease control rate (DCR), defined as the percentage of patients with CR, PR, or stable disease (SD) as their best response; intracranial ORR (icORR), defined as the percentage of patients that achieve intracranial partial response (icPR) or intracranial complete response (icCR) and DoR of intracranial response (icDoR) using equivalent definition to DoR for intracranial response; progression-free survival (PFS), defined as the time elapsed from the date of first dose of encorafenib plus binimetinib to date of disease progression per RECIST v1.1 or death, whichever occurs first; intracranial PFS (icPFS), defined as time from the date of first dose of encorafenib plus binimetinib to date of intracranial disease progression per RECIST v1.1 or death, whichever occurs first; and overall survival (OS), defined as the time from the start of treatment until the date of death due to any cause. For time-dependent endpoints, patients who were lost to follow-up or had no events by the time of the analysis were censored at the date of the last alive contact. Safety was assessed based on the frequency and severity of adverse events (AEs). AEs were codified using the Medical Dictionary for Regulatory Activities (MedDRA), and their severity was categorized according to the Common Terminology Criteria for Adverse Events (CTCAE) v5.0. Treatment compliance was measured by the duration of encorafenib plus binimetinib treatment and the proportion of patients requiring dose interruptions, dose reductions, or permanent treatment discontinuation.

### Statistical Analysis

This descriptive and exploratory study included all identified patients with BRAF^V600^-mutated MBM. The number of participating sites was selected to ensure adequate geographic coverage.

Statistical analyses were performed using descriptive statistics, such as median and their corresponding 95% confidence intervals (CIs) for quantitative variables and frequency and percentages for categorical variables. Response rates were reported as the number and percentage of patients, and their respective 95% CIs were estimated using the Clopper-Pearson method. Time-to-event and survival endpoints were analyzed using the Kaplan-Meier method, and risk factor analyses were performed using the Cox proportional hazard model. All statistical analyses were performed using R (version 3.6.3 [2020-02-29] “Holding the Windsock” R Foundation for Statistical Computing, Vienna, Austria). Statistical significance was set at *P* < .05.

## Results

### Patient Characteristics

Between September 2021 and September 2025, 60 patients with MBM treated with encorafenib plus binimetinib were included. The median age at advanced-stage diagnosis was 57 years (full range: 23-85), and 35 patients (58.3%) were male. Most patients had an Eastern Cooperative Oncology Group Performance Status (ECOG PS) of 0-1 (71.7%) and elevated LDH levels (56.7%). MBM were symptomatic in 34 patients (56.7%), with the most common symptoms being motor deficit (55.9%), headache (32.4%), eye disorders (14.7%), and sensory deficit (14.7%) (Supplementary Table S1). At the initiation of encorafenib plus binimetinib, corticosteroids to manage symptoms were administered in 31 patients (51.7%) at a median dose of 46.1 mg/day of prednisolone or equivalent (95% CI, 26.8-53.6) (Table 1).

**Table 1.** Patient characteristics

Characteristic		BECARE-BRAIN *N* = 60 (%)
Median age at advanced stage disease (full range interval); years		57 (23-85)
Sex; *n* (%)	Male	35 (58.3)
	Female	25 (41.7)
ECOG-PS; *n* (%)	0	27 (45.0)
	1	16 (26.7)
	2	8 (13.3)
	3-4	4 (6.7)
	Unknown	5 (8.3)
Metastatic locations other than brain; *n* (%)	Lung	34 (56.7)
	Lymph nodes	33 (55.0)
	Liver	20 (33.3)
Number of brain metastasis; *n* (%)	1	11 (18.3)
	2-5	16 (26.7)
	>5	29 (48.3)
	Multiple not specified	2 (3.3)
	Unknown	2 (3.3)
Symptomatology of MBM; *n* (%)	Symptomatic	34 (56.7)
	Asymptomatic	26 (43.3)
Corticosteroid use; *n* (%)	Yes	31 (51.7)
	No	29 (48.3)
Brain Surgery; *n* (%)	Yes	5 (8.3)
	No	55 (91.7)
Brain RT; *n* (%)	WBRT	17 (28.3)
	SRS/RS	8 (13.3)
	No	35 (58.3)
LDH; *N* (%)	Normal	20 (33.3)
	>ULN	34 (56.7)
	Unknown	6 (10.0)

Abbreviations: ECOG-PS, Eastern Cooperative Oncology Group Performance Status; LDH, lactate dehydrogenase; RS, radiosurgery; RT, radiotherapy; SRS, stereotactic radiosurgery; ULN, upper limit of normal; WBRT, whole brain radiotherapy.

Five patients (8.3%) underwent brain surgery prior to treatment initiation, and radiotherapy was given prior or concurrently to encorafenib plus binimetinib in 25 patients (41.7%) for a median of 8.0 days (95% CI, 5.0-12.0), 8 of them stereotactic radiosurgery (SRS). Radiotherapy was administered prior to encorafenib plus binimetinib in 7 patients (11.7%) and concurrently in 17 patients (28.3%); one not specified.

The median duration of treatment with encorafenib plus binimetinib was 8 months (95% CI, 6.2-9.9). Encorafenib plus binimetinib was the first-line systemic treatment in 52 patients (86.7%), after the debut of metastatic disease with presence of MBM, and second-line treatment after disease progression to ICIs in eight patients (13.3%). Among patients receiving encorafenib plus binimetinib as second-line treatment after ICI, six had MBM from the initial metastatic diagnosis (i.e. before ICI) and two developed MBM after progression to ICI.

### Efficacy

Overall, encorafenib plus binimetinib achieved an ORR of 58.3% (95% CI, 44.9-70.9), with three patients (5.0%) showing a CR and 32 (53.3%) showing a PR as their best response (Figure 1). Responses were achieved after a median of 2.4 months (95% CI, 2.1-3.1) and lasted for a median DoR of 6.5 months (95% CI, 4.2-8.6). The ORR was 59.6% (95% CI, 45.1-73.0) and 50% (95% CI, 15.7-84.3) in patients treated with first- and second-line encorafenib plus binimetinib, respectively. The icORR was 46.7% (95% CI, 33.7-60.0), with 3 patients (5%) achieving an icCR. The median icDoR was 8.3 months (95% CI, 3.5-11.9) (Supplementary Figure S1).

**Figure 1.**
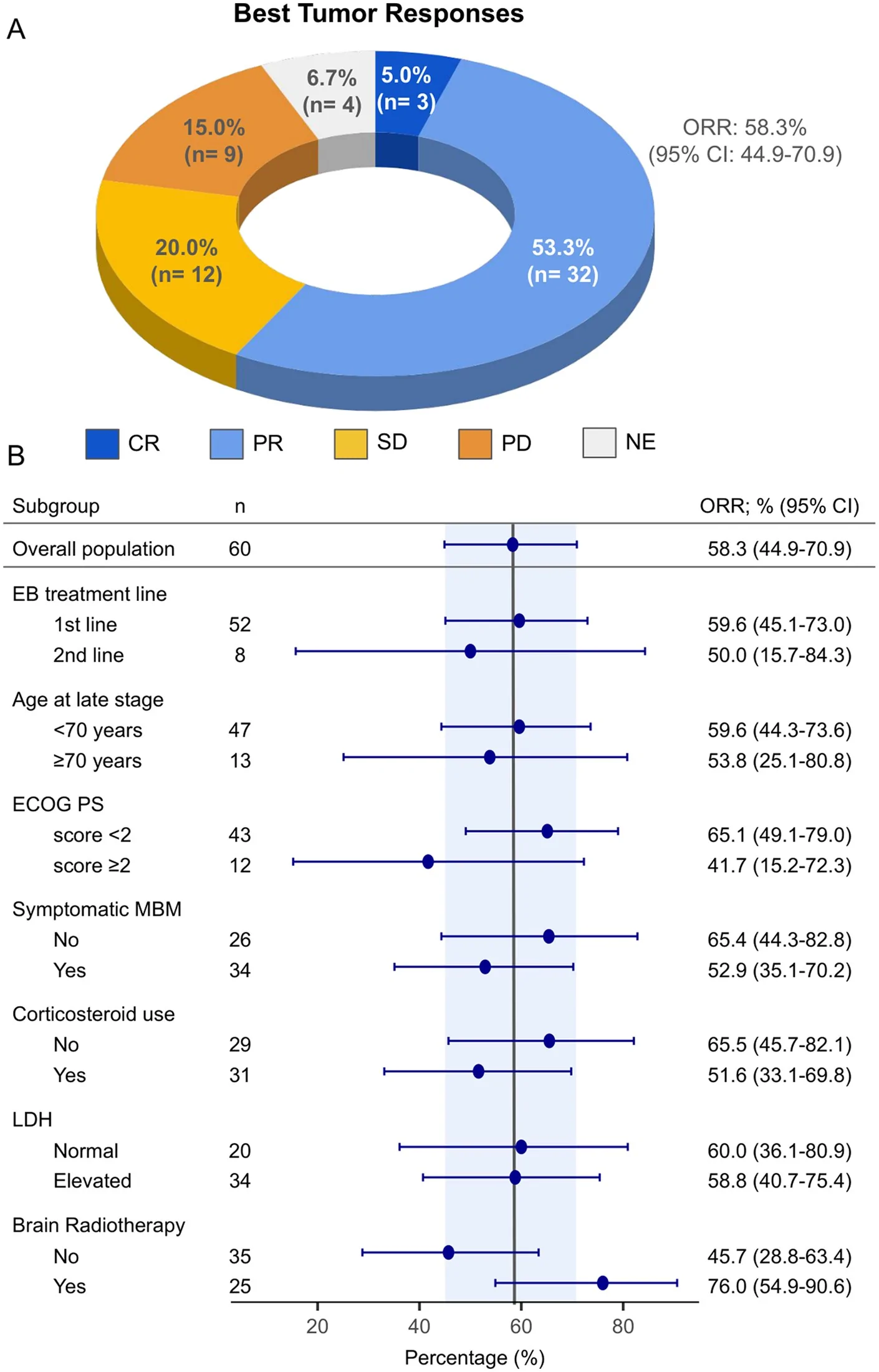
Antitumour activity of encorafenib plus binimetinib. (A) Pie chart illustrating best tumor response rates and ORR rates in the full dataset (*n* = 60). (B) Forest plot illustrating the ORR in the patient subgroups (dots). Whiskers indicate 95% CIs. The vertical line represents the ORR in the full dataset and the area represents its 95% CI. Abbreviations: CI, confidence interval; CR, complete response; EB, encorafenib plus binimetinib, ECOG PS, Eastern Cooperative Oncology Group performance status; LDH, lactate dehydrogenase; MBM, melanoma brain metastasis, Ne, not evaluable; ORR, objective response rate; PD, progressive disease; PR, partial response; SD, stable disease.

Symptomatic patients showed a numerically lower ORR with no significant differences with that of asymptomatic patients (52.9% vs. 65.4%; *P* = .334). Accordingly, the need for corticosteroids showed no significant correlation with a worse ORR (51.6% vs. 65.5%; *P* = .277). Among patients treated with radiotherapy before or concurrently with encorafenib plus binimetinib, the ORR was significantly higher than that in those not receiving radiotherapy (76.0% vs. 45.7%; *P* = .022) (Figure 1B and Supplementary Figure S2). The 8 patients receiving stereotactic radiosurgery (SRS) had tumor responses, 1 CR and 7 PR, accounting for an ORR of 100%.

At the data cutoff, 54 patients (90.0%) had PFS events. The median PFS was 7.4 months (95% CI, 6.2-9.7), with a median follow-up of 10.5 months (95% CI, 7.7-13.6). The 1-year PFS rate was 24.6% (95% CI, 15.6-38.6) (Figure 2). The 1-year PFS rate was 18.4% (95% CI, 10.2-33.1) in patients treated with first-line encorafenib plus binimetinib and 62.5% (95% CI, 36.5-100) in the second-line therapy (Supplementary Figure S3). The median icPFS was 8.2 months (95% CI, 6.8-12.0), with a 1-year icPFS rate of 33.3% (95% CI, 22.7-48.9) (Figure 2).

**Figure 2.**
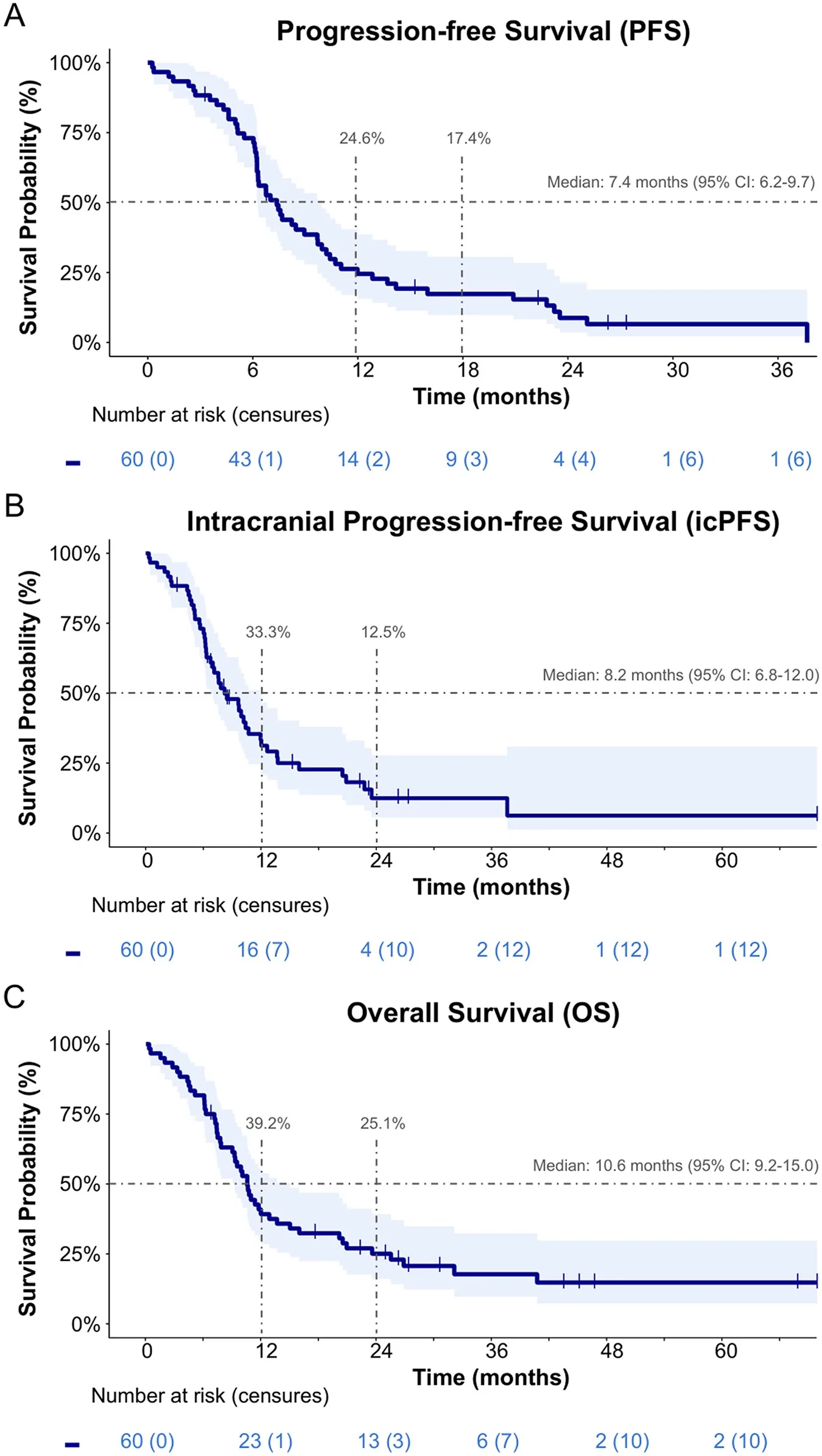
Survival outcomes in the full dataset (*n* = 60). **(**A) Progression-free survival (PFS). (B) Intracranial progression-free survival (icPFS). (C) Overall survival (OS).

Patients receiving short SRS courses of a median 2 days of duration (*n* = 8), 6 of them concurrently to encorafenib binimetinib, showed a median PFS of 11.0 months (95% CI, 8.5-not reached [NR]) (Figure 3). A symptomatic patient treated with corticosteroids for the management of brain symptoms treated with a short course of SRS after the start of encorafenib plus binimetinib reported a PFS time of 37.6 months. The median icPFS for patients with SRS was xXX (Supplementary Figure S4).

**Figure 3.**
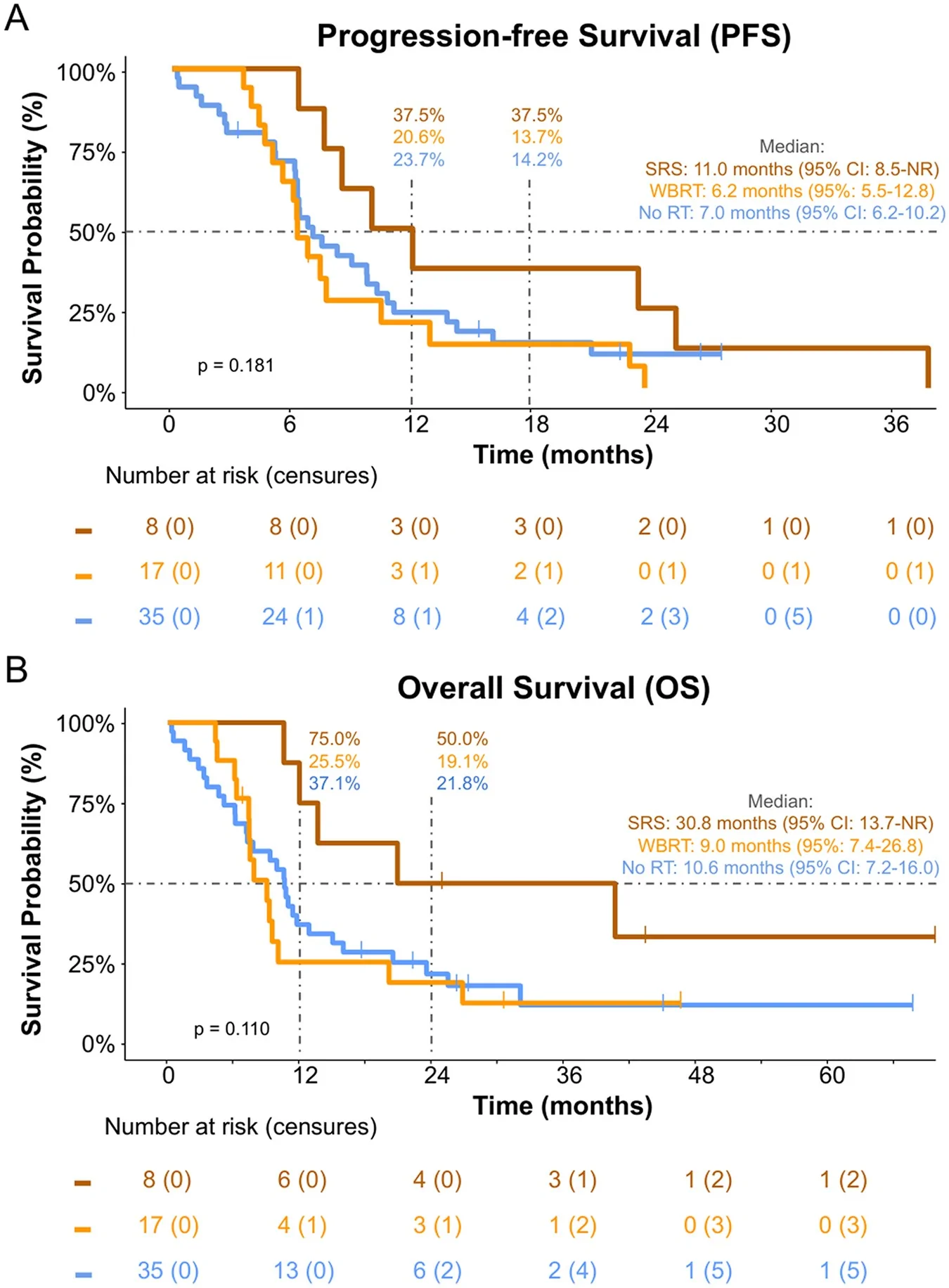
Survival outcomes according to use of concurrent radiotherapy. (A) PFS. (B) OS. Abbreviations: RT, radiotherapy; SRS, stereotactic radiosurgery; WBRT, whole brain radiotherapy.

In total, 48 patients (80.0%) died during the study, all due to disease progression. The 1- and 2-year OS rates were 39.2% (95% CI, 28.5-53.9) and 25.1% (95% CI, 16.0-39.2), respectively, with a median OS of 10.6 months (95% CI, 9.2-15.0) (Figure 2). The 1-year OS rates were 35.6% (95% CI, 24.6-51.5) and 62.5% (36.5-100.0) for patients treated with encorafenib plus binimetinib in the first and second lines of therapy, respectively (Supplementary Figure S3).

Corticosteroid treatment had prognostic value (Supple­mentary Figure S5). The median PFS was 6.2 months (95% CI, 5.1-7.7) vs. 9.9 months (95% CI, 7.6-13.7) for patients with and without corticosteroid treatment, respectively, (HR: 1.6 [95% CI, 0.9-2.7]); and the median OS was 7.7 months (95% CI, 7.1-10.6) and 15.0 months (95% CI, 11.4-40.7), respectively (HR: 2.2 [95% CI, 1.3-4.0]) (Figure 4). The median icPFS was 6.8 months (95% CI, 5.7-16.0) vs. 10.2 months (95% CI, 8.5-13.7) for patients with and without corticosteroid treatment (HR: 1.6 [95% CI, 0.9-2.8]) (Supplementary Figure S6). Symptoms of MBM at baseline showed no correlation with survival (Supplementary Figure S7).

**Figure 4.**
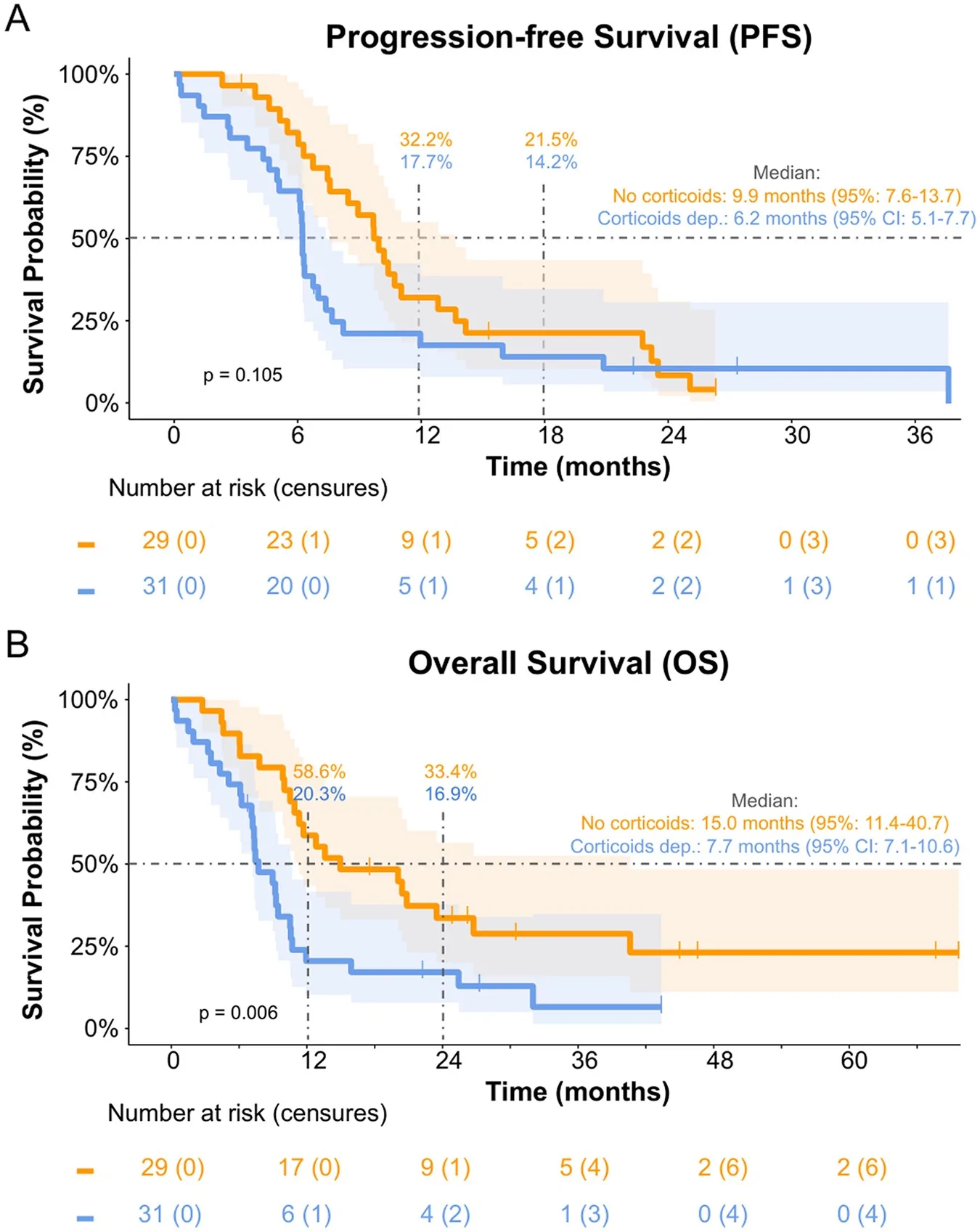
Survival outcomes according to corticosteroid dependency at baseline, before the first dose of encorafenib plus binimetinib. (A) PFS. (B) OS.

### Safety

Treatment-related AEs occurred in 66.7% of patients. The most common treatment-related AEs were diarrhea (*n* = 12; 20.0%), asthenia (*n* = 8; 13.3%), transaminitis (*n* = 7; 11.7%), and constipation (*n* = 6; 10.0%). Diarrhea episodes were grade 1-2 in 11 out of 12 patients experiencing this event. Fever episodes were reported in 5 patients (8.3%), and eye toxicities in 4 patients (6.7%), consisting of grade 1-2 uveitis (3.3%), grade 2 vision decreased (1.7%), and grade 3 retinal vascular disorder (1.7%). Serious AEs were reported in 15 patients (25.0%). Grade ≥3 treatment-related AEs were reported in 12 patients (20.0%), with the most frequent being transaminitis (*n* = 4, 6.7%) and gastrointestinal hemorrhage (*n* = 2, 3.3%) (Figure 5 and Supplementary Table S2). Two patients (3.3%) experienced grade ≥3 hematologic events, consisting of grade 4 febrile neutropenia and grade 3 anemia. Grade 3 hepatitis, ileitis, and hypertension were reported in one patient (1.7%) each.

**Figure 5.**
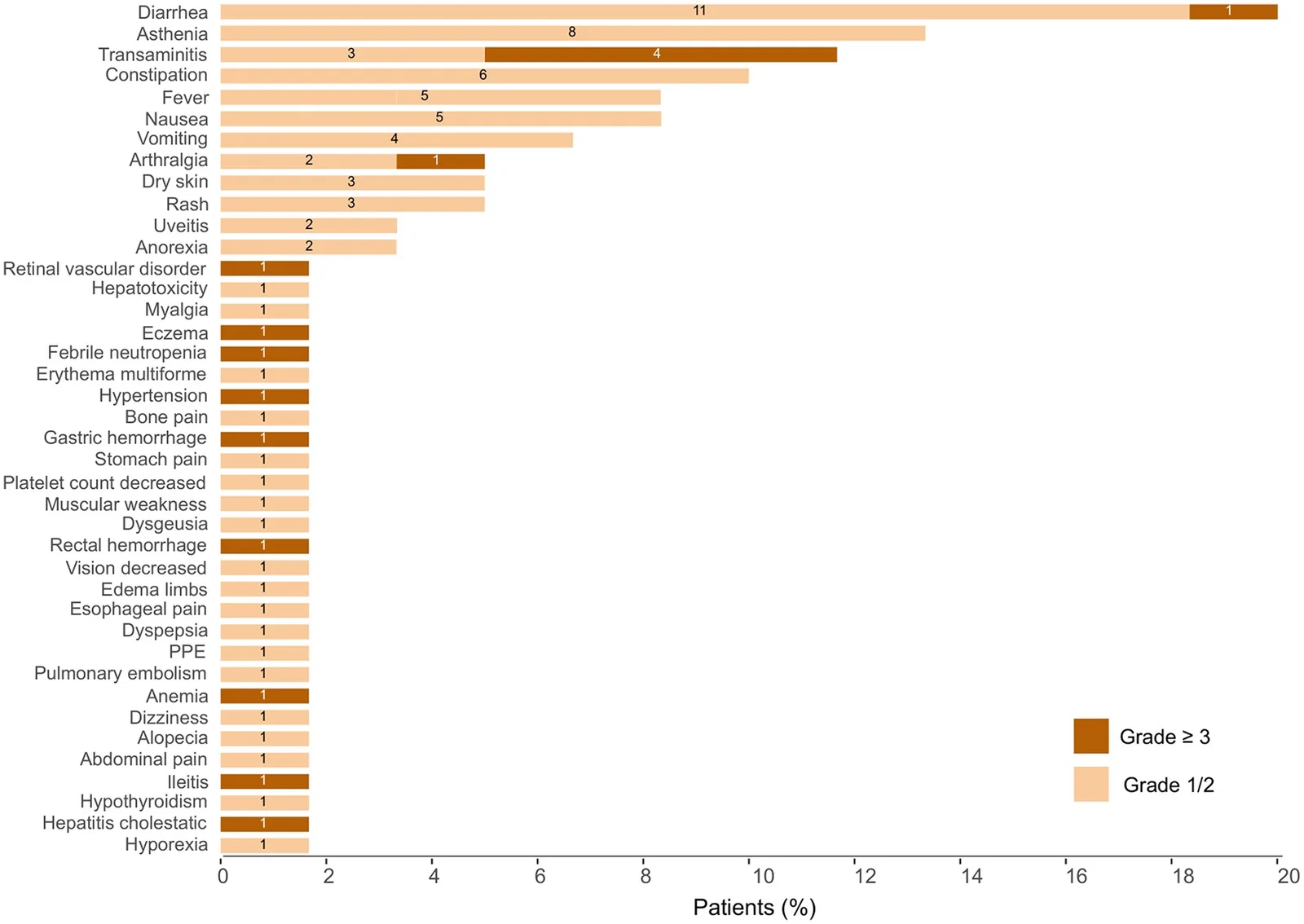
Treatment-related adverse events with encorafenib plus binimetinib in the real world. The bar shows the percentage of patients with events according to grade. The numbers within the bars indicate the number of patients who experienced the corresponding adverse events (*n* = 60).

At the time of the data cutoff, 54 patients (90.0%) discontinued treatment with encorafenib plus binimetinib. The main reasons for treatment discontinuation were disease progression (*n* = 39; 65.0%), death (*n* = 7; 11.7%), toxicities (*n* = 5; 8.3%), investigator criteria (*n* = 2; 3.3%), and loss of follow-up (*n* = 1; 1.7%). Treatment interruptions occurred in 29 patients (48.3%), and dose reductions were required in 19 patients (31.7%). Most dose reductions were undertaken to manage elevation of hepatic enzymes or diarrhea (*n* = 5; 8.5% each), while temporary interruptions were mainly used to handle transaminitis (*n* = 4; 6.7%), fever (*n* = 3; 5.0%), or diarrhea episodes (*n* = 2; 3.3%). No toxic deaths were reported.

## Discussion

BECARE-BRAIN is, to our knowledge, the first real-world study focused on MBM and reporting results from encorafenib plus binimetinib in a large cohort of patients with BRAF^V600^-mutated in Spain.

The ORR (58.3%) was marginally lower than those reported in previous studies.^11^^,^^12^^,^^18^^,^^19^ This discrepancy may be attributable to disparities in patient populations, considering that our cohort comprised a high proportion of patients with multiple brain lesions, elevated LDH levels, symptoms of MBM, or receiving corticosteroid treatment. Our cohort differed substantially from the COLUMBUS trial, in which only nine out of 192 patients (4.7%) had MBM and ECOG-PS >1 was an exclusion criteria.^17^ The median PFS (7.4 months) was numerically superior to that previously documented for encorafenib plus binimetinib in MBM, while the median OS (10.6 months) was consistent with previous studies.^11^^,^^12^^,^^18^^,^^19^ Comparable efficacy outcomes have been observed with other BRAF/MEK inhibitors in real-world.^21-25^ For instance, dabrafenib plus trametinib reported a median PFS of up to 7.2 months for asymptomatic MBM and 4.2 months for symptomatic patients.^10^ Treatment with encorafenib plus binimetinib exhibited encouraging efficacy, particularly given the poor prognosis of the population being studied. Overall, our findings contribute to the growing body of evidence that encorafenib plus binimetinib offers substantial clinical benefits and represents an effective treatment option for BRAF^V600^-mutated MBM.

In subgroup analyses, encorafenib plus binimetinib showed better outcomes for patients with asymptomatic MBM (median PFS 8.2 vs. 6.5 months), as previously described and anticipated, given the worse prognosis in patients with symptomatic disease. However, differences in efficacy between symptomatic and asymptomatic patients were not statistically significant. Conversely, the use of corticosteroids at baseline was significantly associated with worse prognosis (median PFS 6.2 vs. 9.9 months). This suggests that necessity of corticosteroids for managing MBM symptoms may be a determinant of unfavorable outcomes and should be considered when defining symptomatic disease, rather than relying solely on symptoms, as previously suggested.^26^^,^^27^ Furthermore, corticosteroid dependency is a more quantifiable and objective trait that is easier to standardize than patient-reported symptoms of brain disease.

Intracranial and extracranial responses were observed across subgroups, including patients with symptoms and concomitant corticosteroid use. This observation is noteworthy, given that corticosteroid use often poses a limitation to ICI therapy. Considering the caveats of cross-trial comparisons, our results (ORR = 52.9%) compared favorably with those of ICI in patients with symptomatic disease or corticosteroid dependance. Nivolumab and ipilimumab showed response rates of 22%-32% and a median PFS of 1.2-2.7 months in symptomatic patients.^7^^,^^25^ Therefore, encorafenib plus binimetinib is a valid treatment option for symptomatic or corticosteroid-dependent patients, and careful patient selection is recommended to optimize treatment outcomes.

The high survival rates reported in clinical trials and observational studies^7^^,^^25^ may still justify prioritizing ICIs regardless of symptoms and relegate BRAF/MEK inhibitors for ulterior treatment. However, there is no prospective randomized evidence of superiority, and patients with symptoms and requiring corticosteroid treatment remain a challenging scenario. Encorafenib plus binimetinib showed similar or even better efficacy after progression to ICI therapy (median PFS 13.5 vs. 7 months in second- and first-line therapy, respectively). Previous real-world studies reported a similar effect in advanced settings and in patients with MBM.^19^^,^^20^ Therefore, encorafenib and binimetinib seem an effective treatment option even after ICI failure.

Patients receiving prior or concurrent brain radiotherapy showed high response rates (76.0%), as previously reported.^12^ The ORR in patients receiving SRS was 100%. Consistently, prolonged survival has been shown with other BRAF/MEK inhibitors when administered after previous local brain metastases treatment (including radiotherapy).^10^ In the E-BRAIN study, sequential radiotherapy after two months of treatment with encorafenib plus binimetinib was used for selected patients to enhance tumor responses, and an icORR of 70.8% was reported, with most patients experiencing long-lasting responses and a median icPFS of 8.5 months.^12^ Retrospective evidence also showed an independent improvement in survival outcomes for patients receiving SRS.^28^ Additionally, no safety concerns were raised by the combination in our cohort. Based on prior evidence and our findings, the use of SRS should be considered concurrently with encorafenib plus binimetinib, when feasible. Prospective, randomized validation will be required to confirm our observations.

We also observed a substantial proportion of patients receiving whole brain radiotherapy (WBRT), which is often discouraged and only recommended for patients with very disseminated intracranial disease. The use of WBRT may have been motivated by the poor prognosis of our cohort, which had predominantly multiple brain lesions (78.3%), symptomatic disease (56.7%), and corticosteroid dependency (51.7%).

Other studies have explored novel combinations. The TRICOTEL study explored the triplet of atezolizumab, vemurafenib, and cobimetinib in 65 patients with BRAF^V600^-mutated MBM, reporting an ORR of 54 %, median PFS of 5.5 months, and OS of 13.7 months.^29^ Despite promising icORR rates and an acceptable safety profile, triplet combinations are often not exempt from increased toxicity rates.^30^^,^^31^ The combination of relatlimab and ipilimumab showed intracranial activity in patients with MBM.^32^ The TRINITY trial will provide further evidence of the triplet regimen of relatlimab, ipilimumab, and nivolumab, including patients with asymptomatic MBM.^33^ Additionally, the optimal sequentiality of the treatments was explored. The sandwich approach, consisting of short BRAF/MEK induction followed by ICIs and rechallenge with BRAF/MEK inhibitors after disease progression, in the SECOMBIT trial, showed potential protective effects against the development of MBM that were not observed with other sequential treatment combination in the ImmunoCobiVem trial.^16^^,^^34^ These findings were suggestive of potential intracranial activity, and the sandwich of encorafenib plus binimetinib followed by cemiplimab plus fianlimab and rechallenge with encorafenib plus binimetinib is currently being explored by the GEM group in the phase 2 ENCEFALO clinical trial (NCT06887088).^35^

The safety profile of encorafenib plus binimetinib was consistent with that reported in previous studies.^12^^,^^17^ No new safety signals were identified. Most gastrointestinal toxicities were mild in nature. Pyrexia was reported in 8.3% of patients, all grade 1-2. Altogether, the proportion of patients experiencing toxicities was largely in line with those observed with encorafenib plus binimetinib or other BRAF-MEK inhibitors in clinical trials with more active and exhaustive monitoring.^10^^,^^12^^,^^17^

The study faced certain limitations, including the absence of a comparative arm and retrospective data collection, which complicated the interpretation of causality and might have overlooked selection bias. The extensive national territorial coverage and systematic inclusion of cases were strengths that enhanced representativeness of the real-world scenario.

In conclusion, encorafenib plus binimetinib had a manageable safety profile in the real world and showed clinically significant benefits in patients with BRAF^V600^-mutant MBM, especially in patients treated with concurrent corticosteroids for the management of brain symptoms. The potential role of radiotherapy should be explored in prospective trials.

## Supplementary Material

vdag252_Supplementary_Data

## Data Availability

The raw data is protected and not available according to data privacy laws. The data that support the findings of this study are available from the corresponding author upon reasonable request (equivalent to the purposes for which the patients granted their consent to use the data). Data will be provided anonymously, with no identifiable data.
