# Supplementary material for: Encorafenib plus binimetinib in patients with BRAF-mutated melanoma brain metastases: Results from the BECARE (GEM-2002) trial
Source: Neurooncol Adv. 2026 Sep 21;8(1):vdag252. doi: 10.1093/noajnl/vdag252 (PMC13648201; doi:10.1093/noajnl/vdag252)
Supplement: Supplementary file 1 [file vdag252_supplementary_data.pdf]

SUPPLEMENTARY INFORMATION

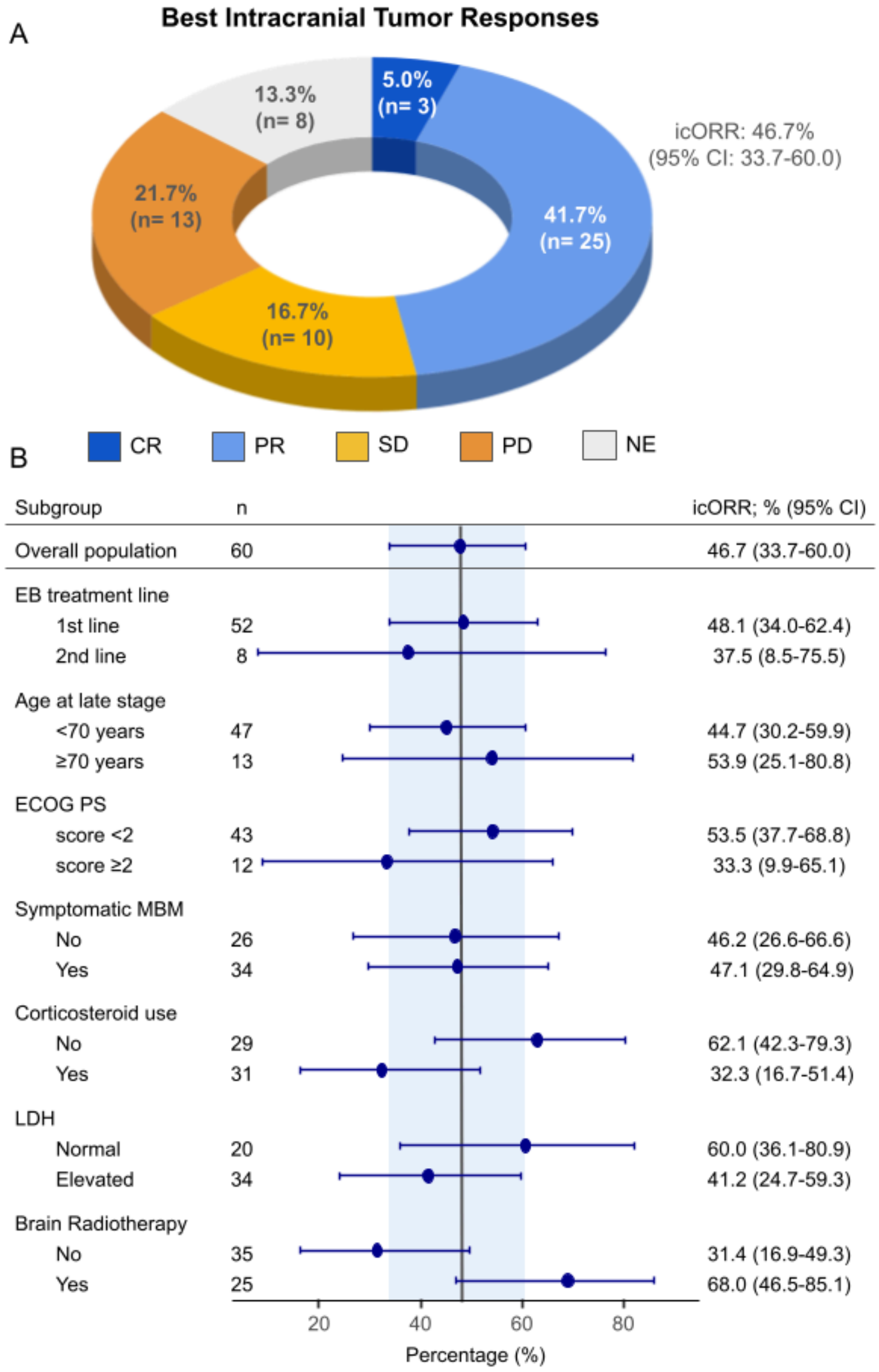

Figure S1. Intracranial antitumour activity of encorafenib plus binimetinib. A) Pie chart illustrating best tumor response rates and icORR rates in the full dataset (n=60). B) Forest plot illustrating the icORR in the patient subgroups (blue dots). Whiskers indicate 95% CIs. The vertical line represents the ORR in the full dataset and the blue area represents its 95% CI.

Abbreviations: CI, confidence interval; CR, complete response; EB, encorafenib plus binimetinib, ECOG PS, Eastern Cooperative Oncology Group performance status; icORR, intracranial objective response rate; LDH, lactate dehydrogenase; MBM, melanoma brain metastasis, Ne, not evaluable; PD, progressive disease; PR, partial response; SD, stable disease.

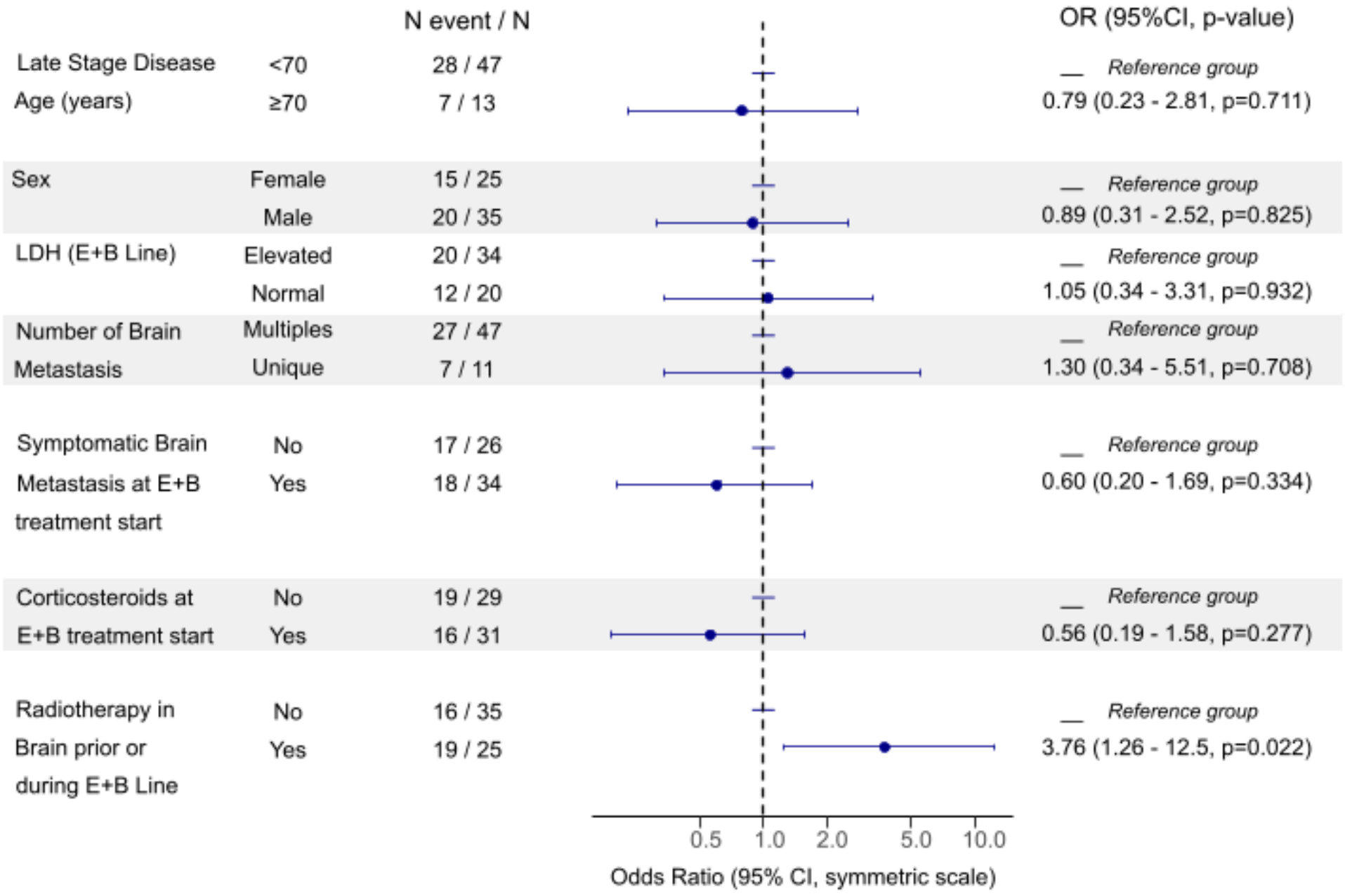

Figure S2. Univariable subgroup analysis of Objective Response Rate (ORR). Dots represent the hazard ratio, calculated comparing to the reference group and the whiskers the 95% CI. Age at late stage disease was the patient age at diagnosis of unresectable stage III or stage IV melanoma.

Abbreviations: CI, confidence interval; E+B, encorafenib plus binimetinib; OR, odds ratio.

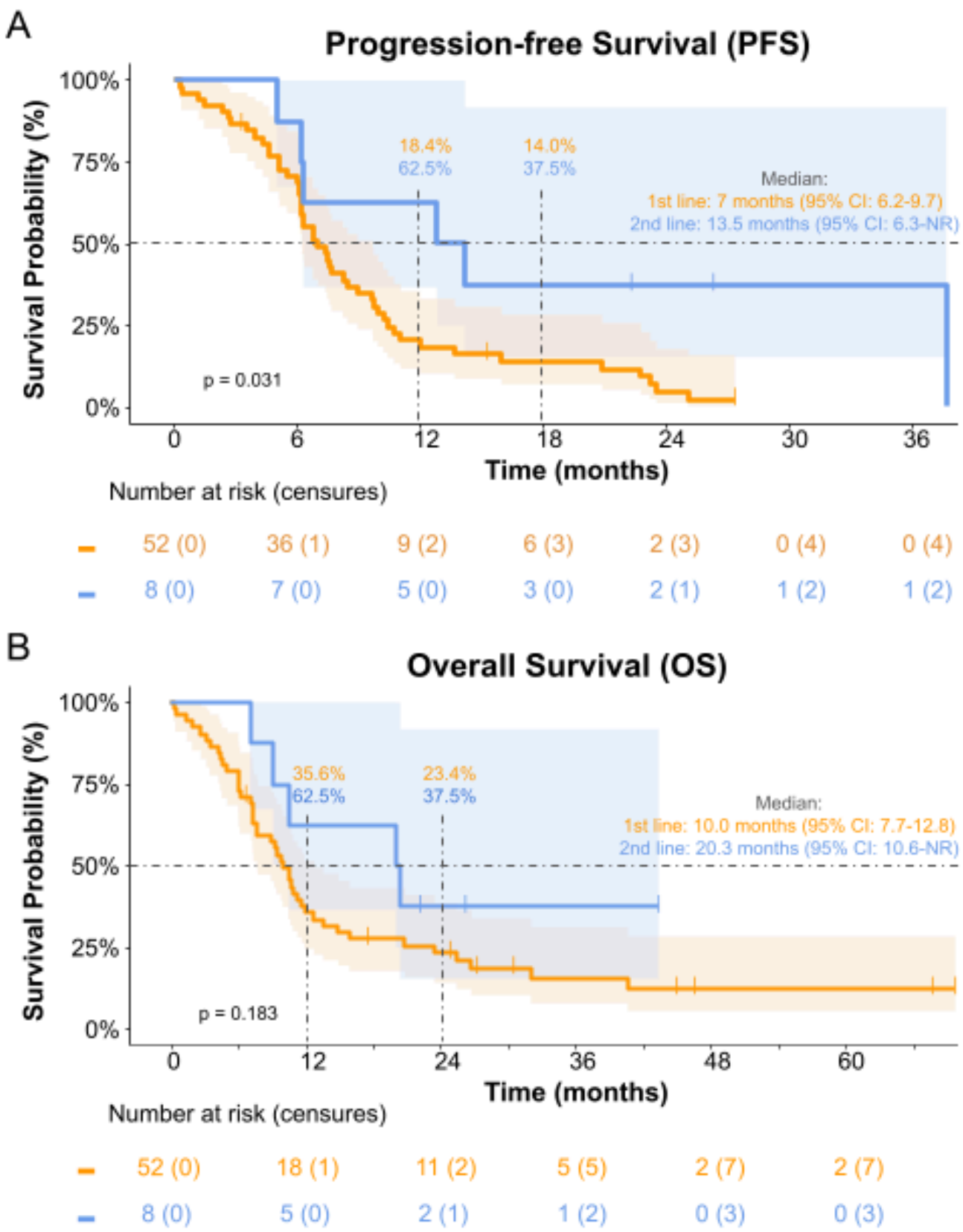

Figure S3. Survival outcomes according to the number of systemic treatment lines in advanced/metastatic setting. A) Progression-free Survival B) Overall Survival.

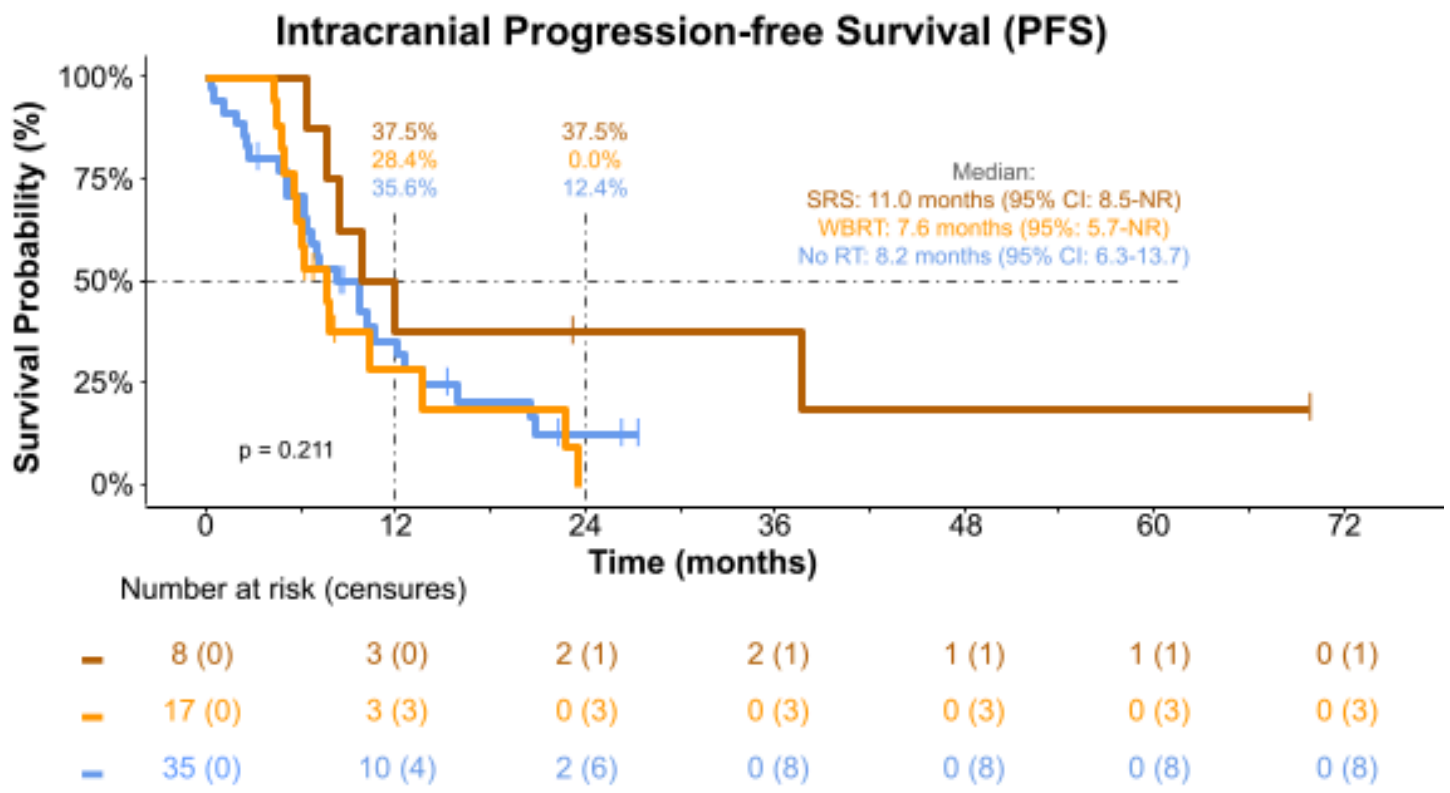

Figure S4. Intracranial PFS according to use of concurrent radiotherapy.

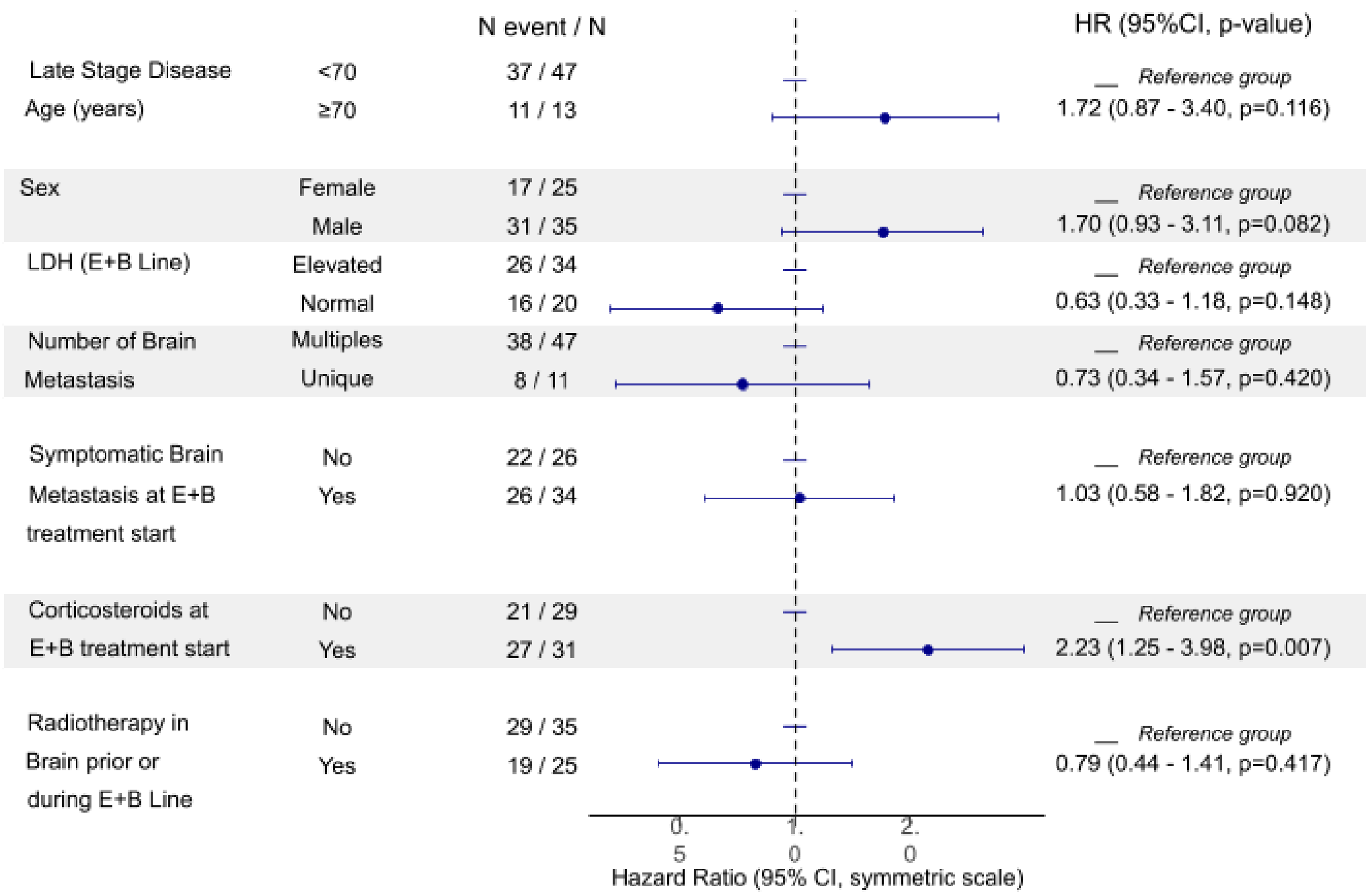

Figure S5. Univariable subgroup analysis of OS. Dots represent the hazard ratio, calculated compared to the reference group and the whiskers the 95% CI.
Abbreviations: CI, confidence interval. E+B, encorafenib plus binimetinib; HR, hazard ratio.

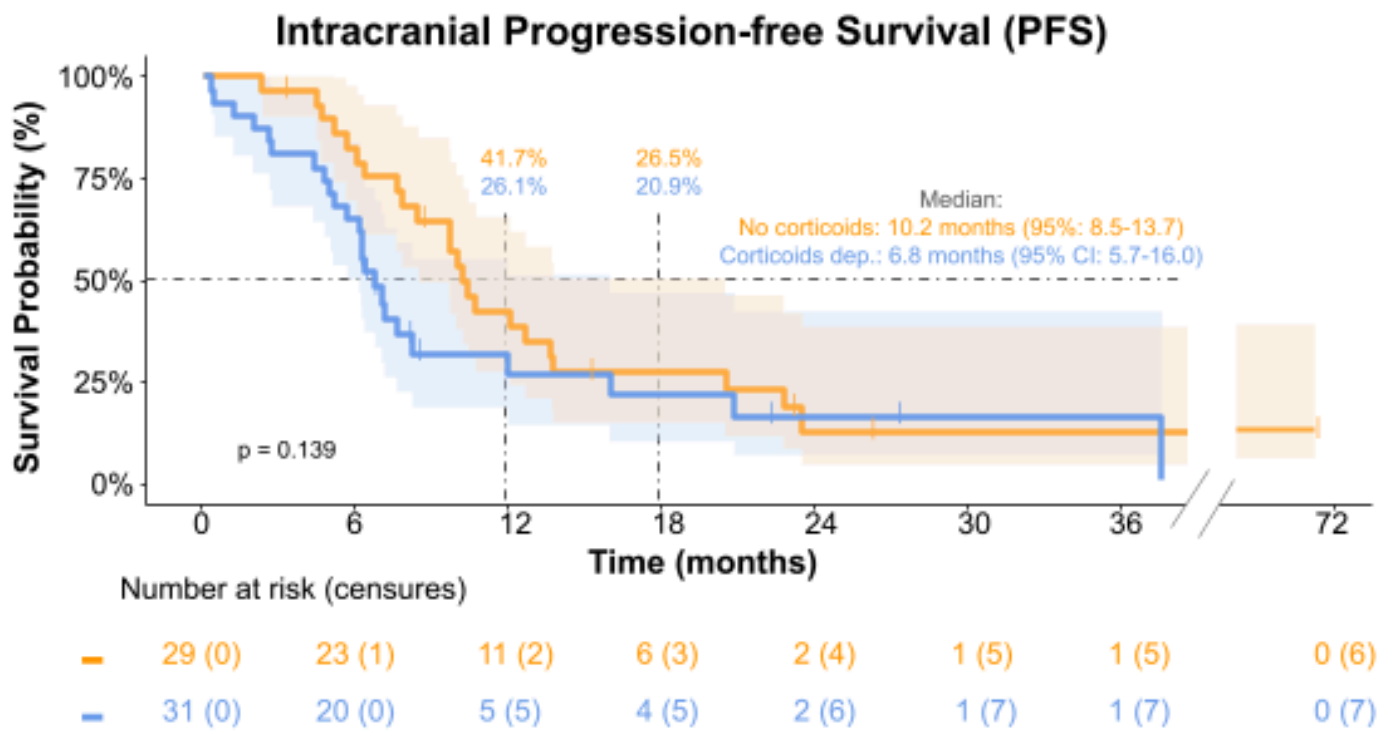

Figure S6. Intracranial PFS according to corticosteroid dependency at baseline, before the first dose of encorafenib plus binimetinib.

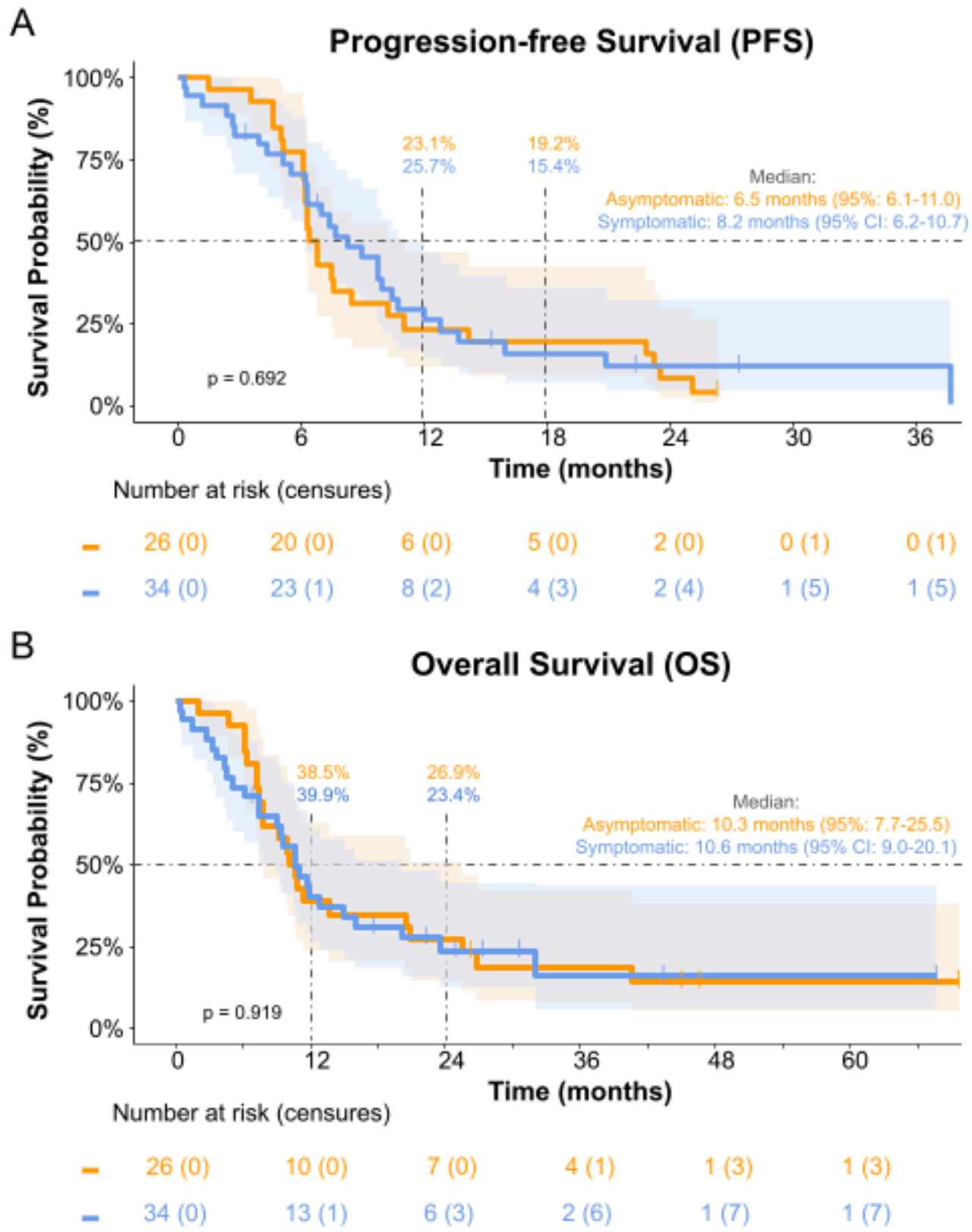

Figure S7. Survival outcomes according to the presence of Melanoma Brain Metastasis (MBM) symptoms at baseline, before the first dose of encorafenib plus binimetinib. A) PFS B) OS.

Table S1. MBM symptoms at the initiation of encorafenib plus binimetinib treatment. Some patients may have experienced more than one symptom.

Symptom	n (%)
Motor deficit	19 (55.9)
Headache	11 (32.4)
Eye disorders [1]	5 (14.7)
Sensory deficit	5 (14.7)
Speech deficit	4 (11.8)
Nausea	3 (8.8)
Epilepsy	3 (8.8)
Musculoskeletal/connective tissue disorder	2 (5.9)
Vomits	2 (5.9)
Asthenia	2 (5.9)

[1] refers to neurological/ophthalmological symptoms directly caused by intracranial lesions or raised intracranial pressure prior to treatment initiation (e.g., blurred vision, diplopia, visual field defects, or papilledema).

Table S2. Safety profile of encorafenib plus binimetinib. Treatment-related adverse events reported in the full dataset (n=60). Events with unknown grades are assumed to be grade 1-2.

Toxicities	Grade 1-2	Grade 3-4	Overall
Diarrhea	11 (18.33%)	1 (1.67%)	12 (20%)
Asthenia	8 (13.33%)	0 (0.00%)	8 (13.33%)
Transaminitis	3 (5.00%)	4 (6.67%)	7 (11.67%)
Constipation	6 (10.00%)	0 (0.00%)	7 (11.67%)
Fever	5 (8.33%)	0 (0.00%)	5 (8.33%)
Nausea	5 (8.33%)	0 (0.00%)	5 (8.33%)
Vomiting	4 (6.67%)	0 (0.00%)	4 (6.67%)
Arthralgia	2 (3.33%)	1 (1.67%)	3 (5%)
Dry skin	3 (5.00%)	0 (0.00%)	3 (5.00%)
Rash	3 (5.00%)	0 (0.00%)	3 (5.00%)
Uveitis	2 (3.33%)	0 (0.00%)	2 (3.33%)
Anorexia	2 (3.33%)	0 (0.00%)	2 (3.33%)

Retinal vascular disorder	0 (0.00%)	1 (1.67%)	1 (1.67%)
Hepatotoxicity	1 (1.67%)	0 (0.00%)	1 (1.67%)
Myalgia	1 (1.67%)	0 (0.00%)	1 (1.67%)
Eczema	0 (0.00%)	1 (1.67%)	1 (1.67%)
Febrile neutropenia	0 (0.00%)	1 (1.67%)	1 (1.67%)
Erythema multiforme	1 (1.67%)	0 (0.00%)	1 (1.67%)
Hypertension	0 (0.00%)	1 (1.67%)	1 (1.67%)
Bone pain	1 (1.67%)	0 (0.00%)	1 (1.67%)
Gastric hemorrhage	0 (0.00%)	1 (1.67%)	1 (1.67%)
Stomach pain	1 (1.67%)	0 (0.00%)	1 (1.67%)
Platelet count decreased	1 (1.67%)	0 (0.00%)	1 (1.67%)
Muscular weakness	1 (1.67%)	0 (0.00%)	1 (1.67%)
Dysgeusia	1 (1.67%)	0 (0.00%)	1 (1.67%)
Rectal hemorrhage	0 (0.00%)	1 (1.67%)	1 (1.67%)
Vision decreased	1 (1.67%)	0 (0.00%)	1 (1.67%)
Edema limbs	1 (1.67%)	0 (0.00%)	1 (1.67%)
Esophageal pain	1 (1.67%)	0 (0.00%)	1 (1.67%)
Dyspepsia	1 (1.67%)	0 (0.00%)	1 (1.67%)
Palmar-plantar erythrodysesthesia syndrome	1 (1.67%)	0 (0.00%)	1 (1.67%)
Pulmonary embolism	1 (1.67%)	0 (0.00%)	1 (1.67%)
Anemia	0 (0.00%)	1 (1.67%)	1 (1.67%)
Dizziness	1 (1.67%)	0 (0.00%)	1 (1.67%)
Alopecia	1 (1.67%)	0 (0.00%)	1 (1.67%)
Abdominal pain	1 (1.67%)	0 (0.00%)	1 (1.67%)
Ileitis	0 (0.00%)	1 (1.67%)	1 (1.67%)
Hypothyroidism	1 (1.67%)	0 (0.00%)	1 (1.67%)
Hepatitis cholestatic	0 (0.00%)	1 (1.67%)	1 (1.67%)
Hyporexia	1 (1.67%)	0 (0.00%)	1 (1.67%)
